# Multidrug resistance protein 4/ ATP binding cassette transporter 4: a new potential therapeutic target for acute myeloid leukemia

**DOI:** 10.18632/oncotarget.2425

**Published:** 2014-09-02

**Authors:** Sabrina Copsel, Ariana Bruzzone, Maria May, Julien Beyrath, Victoria Wargon, Jeannette Cany, G.M. Russel Frans, Carina Shayo, Carlos Davio

**Affiliations:** ^1^ Instituto de Biología y Medicina Experimental-CONICET, Buenos Aires, Argentina; ^2^ Laboratorio de Farmacología de Receptores, Facultad de Farmacia y Bioquímica, Universidad de Buenos Aires, Buenos Aires, Argentina; ^3^ Department of Pharmacology and Toxicology, Radboud University Medical Center, Nijmegen, The Netherlands; ^4^ Department of Laboratory Medicine, Radboud University Medical Center, Nijmegen, The Netherlands

**Keywords:** acute myeloid leukemia, MRP4/ABCC4, leukemic stem cells, apoptosis, differentiation.

## Abstract

Less than a third of adults patients with acute myeloid leukemia (AML) are cured by current treatments, emphasizing the need for new approaches to therapy. We previously demonstrated that besides playing a role in drug-resistant leukemia cell lines, multidrug resistance protein 4 (MRP4/ABCC4) regulates leukemia cell proliferation and differentiation through the endogenous MRP4/ABCC4 substrate, cAMP. Here, we studied the role of MRP4/ABCC4 in tumor progression in a mouse xenograft model and in leukemic stem cells (LSCs) differentiation. We found a decrease in the mitotic index and an increase in the apoptotic index associated with the inhibition of tumor growth when mice were treated with rolipram (PDE4 inhibitor) and/or probenecid (MRPs inhibitor). Genetic silencing and pharmacologic inhibition of MRP4 reduced tumor growth. Furthermore, MRP4 knockdown induced cell cycle arrest and apoptosis *in vivo*. Interestingly, when LSC population was isolated, we observed that increased cAMP levels and MRP4/ABCC4 blockade resulted in LSCs differentiation. Taken together, our findings show that MRP4/ABCC4 has a relevant role in tumor growth and apoptosis and in the eradication of LSCs, providing the basis for a novel promising target in AML therapy.

## INTRODUCTION

Acute myeloid leukemia (AML) is a heterogeneous clonal disorder where early hematopoietic cells fail to differentiate and do not undergo programmed cell death or apoptosis. AML is most common in the elderly, but it represents 15-20% of childhood acute leukemias [1]. In the last decades, chemotherapy has been the treatment of choice for AML but one of the major complications is that the current drugs are highly toxic and poorly tolerated, especially by older patients [2]. Furthermore, despite the high-dose chemotherapy, only 20-30% of patients with AML are cured, emphasizing the need for new therapeutical approaches [3].

The study of multidrug resistance proteins (MRPs), also known as members of the C-family of ATP-binding cassette (ABC) drug transporters, was classically focused on their role in cancer chemotherapy, particularly on their ability to confer clinical drug resistance [4]. However, the pathophysiological actions of these proteins are quite diverse, and transport of cytotoxic xenobiotics as a defense mechanism appears not to be the only important evolutionarily conserved function. Moreover, while several members of the ABC family are established as drug transporters, others also mediate transport of intracellular substances that have relevant functions in cancer biology [5]. In particular, MRP4/ABCC4 is the main transporter for cAMP, a relevant signaling molecule that controls cellular proliferation, differentiation, and apoptosis, especially in hematopoietic development [6-8]. Interestingly, MRP4/ABCC4 expression decreases during leukocyte differentiation, promoting cAMP accumulation in differentiated cells [9]. In accordance, comparison of different AML subtypes showed that the highest level of MRP4/ABCC4 is expressed in the least differentiated subtypes [10].

In recent years, emerging evidence suggests that leukemic stem cells (LSCs) lie at the heart of post-treatment relapse and chemoresistance. Thus, the search for effective LSC-directed therapy by targeting their specific properties such as self-renewal, overexpressed cell-surface proteins or aberrant differentiation is a critical challenge [11]. In particular, the increasing recognition that molecules involved in stem cell differentiation have a key role in carcinogenesis prompts our investigation in this direction. Previous studies have shown that various ABC transporters are highly expressed in LSCs as part of their self-protection capabilities [12].

In the last years our research has been focused on the regulation of cAMP levels with potential implication in the differentiation and/or cytotoxicity of hematopoietic cancer cells. In this regard, we reported that MRP4/ABCC4 regulates intracellular cAMP levels in AML cell lines and contributes to cell proliferation and differentiation [13]. Based on these findings, the aim of the present work was to advance into the knowledge of MRP4/ABCC4 as a potential new therapeutic target for AML. For this purpose, we evaluated the role of this transporter in tumor progression in a mouse xenograft model and in the temporal resolution of cAMP response in LSCs differentiation. Here, we show that MRP4/ABCC4 blockade strongly reduced tumor growth inducing cell cycle arrest and apoptosis *in vivo*. In addition, we found that increased cAMP levels and MRP4/ABCC4 inhibition resulted in LSCs differentiation. Altogether, these findings show that MRP4/ABCC4 has a relevant role in tumor growth and apoptosis and in the eradication of LSCs, providing the basis for a novel promising target in leukemia therapy.

## RESULTS

### Phosphodiesterase 4 (PDE4) and MRPs inhibition decrease tumor growth inducing cytostasis and apoptosis in an AML *in vivo* model

**Figure 1:**
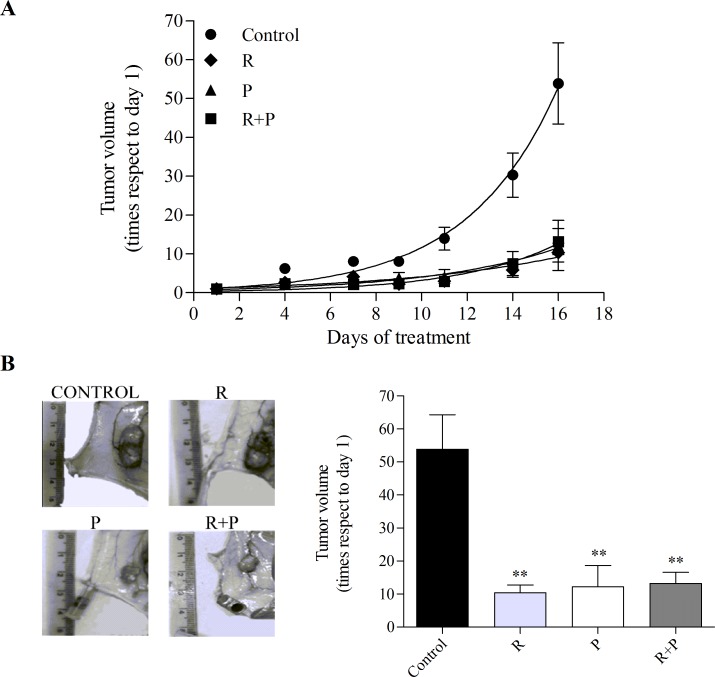
Effect of MRP and PDE4 inhibition on mouse AML tumor volume Nude mice were subcutaneously injected with U937 AML cells. When tumors were palpable, animals (six per group) were treated with vehicle (control), rolipram (R,1.5 mg/kg), probenecid (P, 50 mg/kg), or in combination (R+P) for 16 days. (A) Growth curves. Tumors were measured every 2 or 3 days and volumes calculated as described in “Materials and methods”. (B) Representative picture of the dissected tumors of each group (*left panel*) and tumor volumes (*right panel*) at the end of the experiment. Data represent mean±SEM. **, p<0.01 vs. control. A representative experiment of the other two is plotted.

Cyclic AMP levels have been associated with leukemia cell proliferation. We have recently reported that intracellular cAMP accumulation induced by PDE4 and MRP inhibition significantly decreases cell proliferation in diverse AML cell lines [13]. Thus, to evaluate the effect of cAMP regulation on leukemia cell proliferation *in vivo*, a human AML model was established by subcutaneously injecting Swiss nu/nu mice with U937 cells. When tumors were palpable, mice were treated with intraperitoneal injections of probenecid (MRP inhibitor, 50 mg/kg), rolipram (PDE4 inhibitor, 1.5 mg/kg), probenecid+rolipram or with vehicle 5 times per week for 2 weeks. Tumors were measured 3 times per week and volumes calculated. Interestingly, all treatments showed inhibition of tumor growth, a significant smaller tumor volume was achieved in probenecid and/or rolipram-treated animals as compared with vehicle-treated mice after 2 weeks (P<0.01; Figure 1A-B).

**Figure 2:**
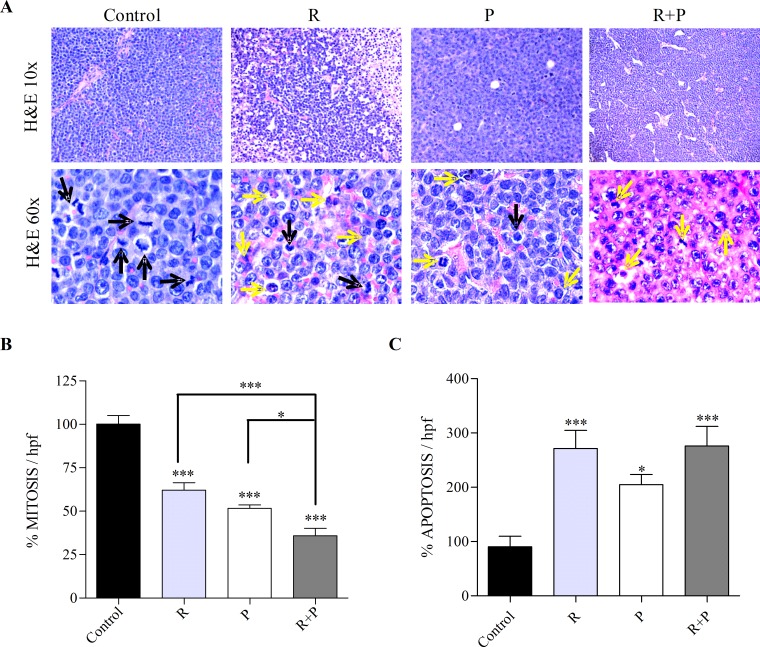
Tumor morphology and quantification of mitosis and apoptosis in rolipram or/and probenecid treated-mice Tumors from mice treated with rolipram (R), probenecid (P), or both (R+P) were processed for histological evaluation. (A) Representative images of H&E-stained tumor slides are shown. Mitotic images (black arrows) and apoptosis (yellow arrows) are displayed in the H&E stained slides. The number of mitotic (B) and apoptotic (C) cells was counted in the H&E stained sections, using 600x magnification, by direct evaluation of 10 and 15 high power fields (HPF), respectively. The mean value ±SEM obtained in control slides was considered as 100%. *, p<0.05; ***, p<0.001.

Next, the tumor morphology was evaluated in H&E-stained sections, with emphasis on tissue organization as a whole (evaluated at low magnification), the presence of neo-vascularization (new, heterogeneous, one layer endothelial vessels), the cytological characteristics of tumor cells, mitosis and apoptosis. Control tumors exhibited a solid monomorphic sheet with no necrotic or hemorrhagic areas, while treated tumors showed variable necrotic areas (Figure 2A). A significant decrease in the mitotic index was observed in mice treated with rolipram, probenecid, and rolipram with probenecid. Moreover, the latter treatment was even more effective in decreasing the number of mitosis compared with mice treated with rolipram or probenecid alone (Figure 2B). In addition, as shown in Figure 2C, a significant increase in the apoptotic index was observed in mice treated with all compounds. Hence, the mitotic and apoptotic indexes correlated with the effect of PDE4 and/or MRPs inhibition on tumor growth.

### MRP4 knockdown decreases tumor growth *in vivo*

**Figure 3:**
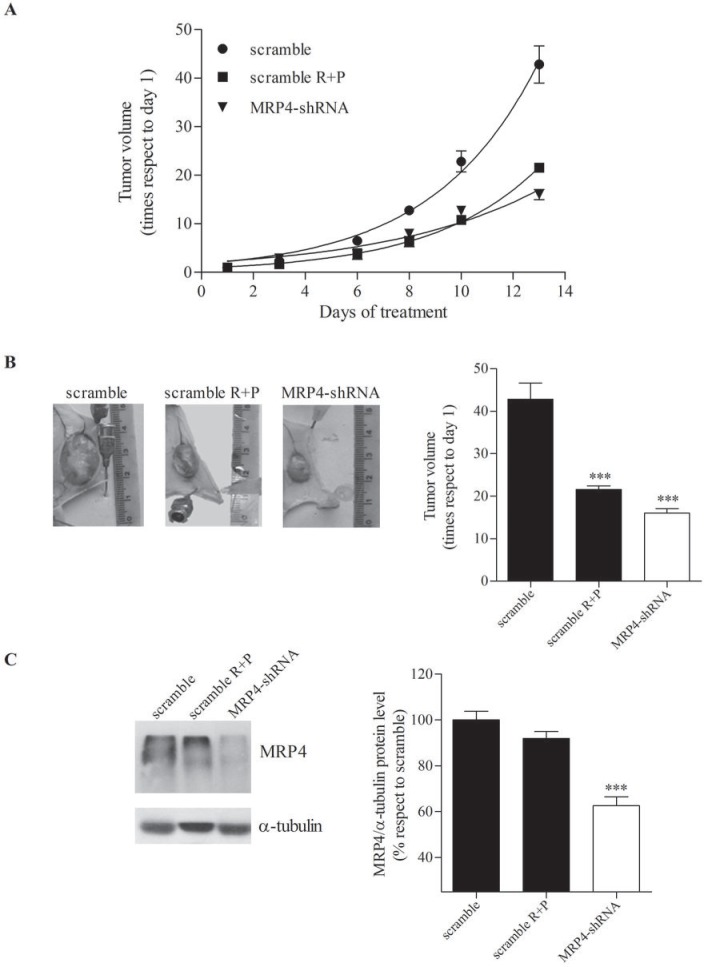
Effect of MRP4 knockdown on mouse AML tumor volume Nude mice were subcutaneously injected with U937 expressing shRNA against MRP4 (MRP4-shRNA) or scrambled shRNA as a control (scramble). A third group of mice, carrying U937-scramble tumors, was treated with rolipram and probenecid (scramble R+P, 1.5 mg/kg and 50 mg/kg, respectively) (A) Growth curves. Tumors were measured every 2 or 3 days and volumes calculated as described in “Materials and methods”. (B) Representative picture of the dissected tumors of each group (*left panel*) and tumor volumes (*right panel*) at the end of the experiment. (C) Representative western blot of protein extracts from scramble, scramble R+P and MRP4-shRNA tumors using specific antibodies against MRP4/ABCC4 and α -tubulin (loading control). The band intensities in different western blots were quantified using ImageJ (US National Institutes of Health). Data represent mean±SEM. ***, p<0.001 vs. control. A representative experiment of the other two is plotted.

As MRP4 is the main transporter for cAMP and this signaling molecule has important implications in hematopoietic cell proliferation we aimed to evaluate whether MRP4 played a role *in vivo* in AML tumor growth. Nude mice were injected with U937 cells expressing short hairpin RNA (shRNA) against MRP4 (MRP4-shRNA) or scrambled, used as a control. The generation and characterization of these cells with stable MRP4-knockdown has been previously reported by us [13] and tested by western blot and cAMP assay just before mice injection (Supplementary Figure 1). In order to compare this molecular approach with the most effective pharmacological treatment (Figure 2), a third group of mice carrying U937-scramble tumor was treated with both rolipram and probenecid (1.5 mg/kg and 50 mg/kg, respectively) 5 times per week for 2 weeks. Remarkably, silencing of MRP4 strongly reduced tumor growth likewise the pharmacological treatment (Figure 3A-B).

To provide further evidence that MRP4 knockdown was effective *in vivo*, following 2 weeks MRP4 expression was assessed by western blot in scramble, rolipram+probenecid and MRP4-shRNA tumors. As expected, a significant reduction in MRP4 protein levels was observed in MRP4-shRNA tumors as compared with scramble tumors. However, MRP4 expression was similar in rolipram+probenecid and scramble tumors, although we have previously reported that rolipram and probenecid increase intracellular cAMP levels [13], (Figure 3C).

### MRP4 knockdown decreases the mitotic index and induces G1 phase arrest by reducing cyclin D1 and increasing p21^Waf1/Cip1^ expressions

**Figure 4:**
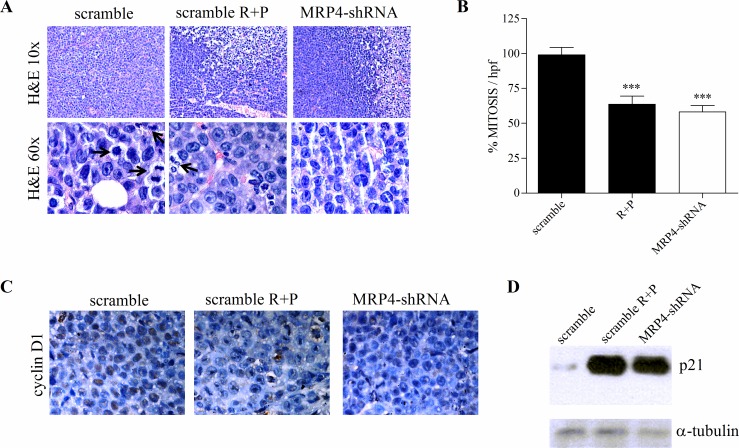
Effect of MRP4 knockdown on tumor morphology, mitotic index and cell cycle arrest Tumors from scramble, rolipram+probenecid (scramble R+P) and MRP4-shRNA groups were processed for histological evaluation, immunohistochemistry and western blot. (A) Representative images of H&E-stained tumor slides are shown. Mitotic images (arrows) are displayed in the H&E 600x slides. (B) The number of mitotic cells was counted in the H&E stained sections, using 600x magnification, by direct evaluation of 10 high power fields (HPF). The mean value ±SEM obtained in control slides was considered as 100%. (C) Immunohistochemistry of paraffin-embedded tumors was performed using an antibody against cyclin D1. Brown corresponds to the antibody signal and blue marks nuclei stained with hematoxilin. (D) Representative western blot of protein extracts from scramble, scramble R+P and MRP4-shRNA tumors using specific antibodies against p21 and α-tubulin (loading control).

After two weeks, tumors were excised and their morphology evaluated in H&E-stained sections. MRP4-shRNA tumors developed vast necrotic areas, and evident neo-vascularization; no significant histological differences were observed between this group and the rolipram+probenecid-treated mice (Figure 4A). Furthermore, tumors from MRP4-shRNA mice showed a significant decreased mitotic index as compared with control mice, although no differences were observed with mice treated with both rolipram and probenecid (Figure 4B).

Next, we assessed *in vivo* the effect of silencing MRP4 or inhibiting MRPs and PDE4 on the expression of key regulators of G1 phase progression including cyclinD1 and p21^Waf1/Cip1^. As shown in Figure 4C, immunohistochemistry analysis revealed a decreased cyclin D1 expression in MRP4-shRNA and rolipram+probenecid tumors compared with the scramble shRNA. In accordance, the levels of p21 exhibited a dramatic up-regulation in both groups (Figure 4D). Collectively, these results indicate that MRP4 blockade or rolipram+probenecid treatment, triggers cytostasis by inducing G1 phase arrest.

### MRP4 knockdown induces apoptosis *in vivo*

**Figure 5:**
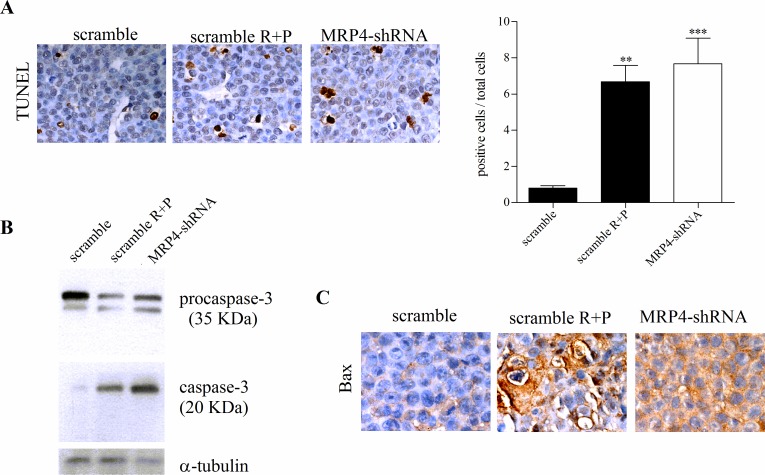
Pro-apoptotic activity of MRP4 knockdown in AML *in vivo* tumors (A) Left. Terminal deoxynucleotidyltransferase (TdT)-mediated dUTP digoxigenin nick-end labelling (TUNEL) of scramble, rolipram+probenecid (scramble R+P) and MRP4-shRNA tumors. *Right.* Quantification of apoptotic cells, stained cells by TUNEL, with respect to total cells per field. Data represent the mean±SEM. n=3 for each tumor type. **, p<0.01and ***, p<0.001vs. scramble. (B) Representative western blot of protein extracts from scramble, scramble R+P and MRP4-shRNA tumors using specific antibodies against caspase-3 and α -tubulin (loading control). Procaspase-3 (35 KDa) and cleaved caspase-3 (20 KDa) are shown. (C) Immunohistochemistry of paraffin-embedded tumors was performed using an antibody against Bax. Brown corresponds to the antibody signal and blue marks nuclei stained with hematoxilin.

Cyclic AMP plays a central role in several cellular processes like proliferation, differentiation and apoptosis [6,8]. The human U937 promonocytic leukemia cell line can be forced to differentiate into monocytes under the appropriate treatment [14]. In a previous paper, we reported that MRP pharmacological inhibition as well as specific MRP4 knockdown induced U937 cell differentiation by increasing cAMP intracellular levels [13]. Therefore, in the present study we evaluated cell differentiation in tumors from mice treated with rolipram and probenecid and with MRP4-shRNA. No significant differences were observed on the expression of the differentiation markers CD11b, CD14, CD88 (data not shown). As cAMP-elevating agents may trigger differentiation and/or apoptosis, we next quantified apoptosis by TUNEL staining. Figure 5A depicts a complete field for tumors from scramble, rolipram+probenecid and MRP4-shRNA groups. In both cases, the number of apoptotic cells was significantly higher than in the scramble shRNA control group (Figure 5A, right graph).

Caspase-3 is a very important regulator of apoptosis in response to a variety of stimuli. In accordance with TUNEL results, the inhibition of MRP and PDE4 as well as MRP4 specific knockdown, induced caspase-3 cleavage-mediated activation (Figure 5B). In line with these findings, an increased expression of Bax, a proapoptotic protein, was detected in the MRP4-shRNA and rolipram+probenecid tumors. However, while MRP4 knockdown tumors presented a uniform staining, the rolipram+probenecid group exhibited heterogeneous stained sections. This difference observed in Bax expression correlates well with the different approaches used to inhibit MRP4 (Figure 5C).

Taken together, these findings show that both pharmacological treatment and MRP4 knockdown induce apoptosis but not cell differentiation in tumors *in vivo.*

### MRP4 is expressed in isolated LSCs

**Figure 6:**
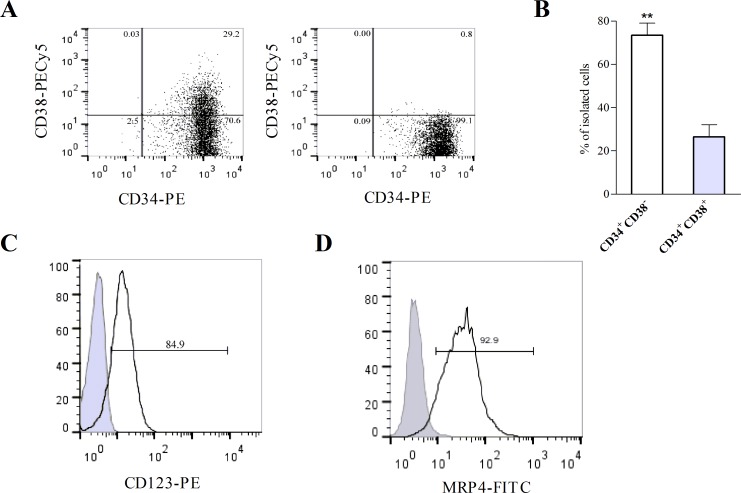
KG-1a cell line contains leukemic stem-like cells expressing MRP4/ABCC4 (A) Flow cytometry analysis of cell surface expression of CD34 and CD38 in KG-1a cells before (*left panel*) and after sorting using FACS (*right panel*). (B) The percentages of CD34+CD38-(LSCs) and CD34+CD38+ (non-LSCs) cells were obtained after independent sortings. **, p<0.01. Data represent the mean±SEM (n=4). (C) Histogram overlay shows CD123 expression in CD34+CD38- cells (black curve) versus IgG1-PE isotype control (filled gray curve). (D) Histogram overlay shows cell surface expression of MRP4/ABCC4 within CD34+CD38-cells (black curve) versus the unspecific control (filled gray curve). Data are from one experiment representative of three.

Results so far indicate that MRP4 is involved in tumor growth and apoptosis, suggesting that MRP4 might be a new target for AML treatment. Based on these observations and the increasing recognition that relapse of AML reflects the failure of current therapies to effectively target leukemic stem cells (LSCs), we aimed to isolate LSCs in the U937 AML cell line. However, in these cells the LSC population was less than 0.1%, supporting that the U937 cell line is not a good model to study LSCs (data not shown). It was recently reported that KG-1a, a human undifferentiated AML cell line, is a suitable cellular model to study LSCs [15]. Therefore, in order to confirm the presence and percentage of LSCs (CD34+CD38-) in the KG-1a cell line, cells were stained with a combination of CD34-PE and CD38-PECy5 antibodies and then analyzed by flow cytometry. Results showed that KG-1a cells contained more than 70% of CD34+CD38- cells (Figure 6A, left panel). Then, CD34+CD38- and CD34+CD38+ populations (LSCs and non-LSCs, respectively) were sorted from the cell line using FACS. Post-sorting cell purity revealed that the LSCs-sorted population was 99% pure (Figure 6A, right panel). Four sortings were performed and the percentage of LSCs was always higher than non-LSCs (68±9% vs 26±5%; p<0.01) (Figure 6B). To further characterize the CD34+CD38- sorted population, cells were stained with CD123 antibody given that CD123 (IL-3 receptor α chain) is a unique marker for human AML stem cells [16]. As shown in Figure 6C, a high percentage of the CD34+CD38- sorted population expressed CD123.

AML stem cells have been shown to express various ABC transporters conferring resistance to a broad spectrum of chemotherapeutic drugs [12]. In order to validate MRP4/ABCC4 as a new target for the eradication of LSCs, we assessed the presence of this transporter in the sorted populations. MRP4/ABCC4 protein expression in the CD34+CD38- population was shown by flow cytometry (Figure 6D).

### MRP4/ABCC4 inhibition results in LSCs differentiation

**Figure 7:**
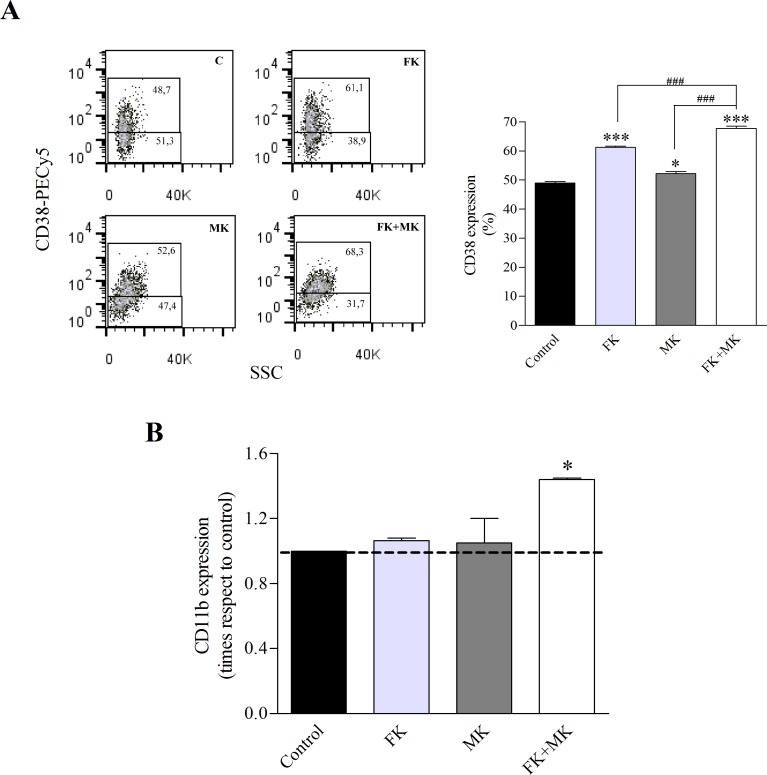
Treatment of LSCs with FK and MK results in LSCs differentiation KG-1a cells were exposed to 10 μM forskolin (FK), 50 μM MK-571(MK) or FK+MK for 48 h. (A) Representative flow cytometry analysis of CD38 expression (*left panel*) and percentage of CD38 positive cells in the LSC population (*right panel*). (B) CD11b antigen expression in the LSC population. *, p<0.05; ***, p<0.001 versus control. ###, p<0.001 versus FK+MK. Data represent mean±SEM (n=3).

Since MRP4/ABCC4 is involved in the regulation of cAMP levels, which are critical for leukemic cell differentiation, we further analyzed its role in LSCs differentiation. First, in order to maintain the LSC population undifferentiated in culture we determined the optimal culture conditions. A marked increase in proliferation rate was observed in CD34+CD38- cells cultured in IMDM compared to the cells in GBGM, a medium developed for the culture of hematopoietic stem cells [17]. However, after 7 days in culture in IMDM, CD34+CD38- cells tended to differentiate into the CD34+CD38+ population, while in GBGM they retained their undifferentiated phenotype (Supplementary Figure 2). Subsequently, we employed GBGM to determine the effect of cAMP modulators and/or MRP4/ABCC4 inhibitor, on CD34+CD38- cells using non-cytotoxic concentrations of the compounds. LSCs differentiation was assessed by CD38 and CD11b expression in the presence of 10 μM forskolin (FK; direct adenylyl cyclase activator), 50 μM MK-571 (MK; MRP4/ABCC4 inhibitor), either alone or in combination, for 48 h. As shown in Figure 7A, FK or MK induced CD38 expression. Moreover, co-incubation with both agents yielded significantly higher levels of this marker.

To gain more insight into LSCs differentiation, the myelomonocytic lineage marker CD11b was evaluated. Neither FK nor MK alone induced expression of this differentiation marker in the LSC population. However, cells treated with both FK and MK enhanced CD11b surface expression (Figure 7B).

Present findings indicate that MRP4/ABCC4 might be a new target for LSCs eradication in AML by inducing their differentiation.

## DISCUSSION

The study of ABC transporters was classically focused on their role in cancer chemotherapy, particularly on their ability to confer resistance to a wide range of chemotherapeutic agents [4]. However, while several members of the ABC family are established as drug transporters, others also mediate transport of endogenous substances that have relevant functions in cancer biology [5]. A number of clinical studies have correlated ABC transporter levels with malignant progression and a more aggressive phenotype. In particular, current evidences suggest that MRP4/ABCC4 is implicated not only in chemotherapy resistance, but also in cancer biology. In this respect, recent studies have shown that MRP4/ABCC4 is overexpressed in several solid tumors such as prostate cancer, non-small cell lung cancer, aggressive primary neuroblastoma, and pancreatic cancer [18-22]. Increased MRP4/ABCC4 levels have been significantly associated with poor clinical outcome in neuroblastoma. It has been reported that MRP4 is expressed in blast cells of patients with AML and higher levels of this protein were detected in the less differentiated FAB subtypes M0 and M1; however, its expression has no influence on treatment outcome using AraC [9,10]. Recently, frequent copy number alterations of MRP4/ABCC4 were observed in *de novo* AML and variable expression of this transporter was detected among AML subtypes from 155 pediatric patients [23]. In addition to cancer, this transporter appears to be involved in the development and progression of pulmonary arterial hypertension, a severe vascular disease [24]. Thus, this transporter has recently emerged as a new promising target for these pathologies.

MRP4/ABCC4 has specificity for a wide range of endogenous substrates. It is the major cAMP efflux transporter, a cyclic nucleotide involved in the regulation of cellular proliferation, differentiation and apoptosis [25,26]. Indeed, previously we demonstrated that besides playing a role in drug-resistant leukemia cell lines, MRP4/ABCC4 regulates leukemia cell proliferation and differentiation through the endogenous MRP4/ABCC4 substrate, cAMP [13]. Unlike other ABC family members, little is known about this transporter and its role in leukemia progression, independent of its drug efflux properties. To our knowledge, present findings provide for the first time strong evidence that MRP4/ABCC4 is involved in tumor growth, differentiation and/or apoptosis in AML.

Leukemic stem cells (LSCs) are crucial in leukemia initiation, progression, metastasis and relapse. Therapeutic approaches targeting mature leukemic cells may clinically improve the disease, although they are unlikely curative if LSCs are not targeted or are resistant to the therapy. Hence, new treatments should be tested on this population. Tumors are the primary source for cancer stem cells (CSCs), although cancer cell lines are attractive alternative sources given that they are not contaminated with normal stem cells [27]. On the other hand, stem cells in tissues are usually in a very small number, which makes it rather difficult to identify and further to isolate them. In this study we detected LSCs (CD34+CD38-) in the undifferentiated AML cell line KG-1a and revealed that they represent approximately 70% of the cell population, whereas in other AML cell lines they are less than 1% and in AML patients around 0.01% to 0.09% [11,28]. Previous studies reported that LSCs isolated from the KG-1a cell line have LSC-specific features, including self-renewal capacity [15]. In accordance, the present study showed that the CD34+CD38- sorted population expresses CD123, a stem cell-specific marker in AML. These findings support that the KG-1a cell line is a suitable model to isolate and study LSCs.

The high expression of ABC transporters involved in the extrusion of chemotherapeutic compounds was proposed to be a major mechanism of drug resistance in LSCs [12]. Interestingly, when different AML subtypes were compared, the least differentiated subtypes expressed the highest level of MRP4/ABCC4 [10]. Moreover, mRNA of this transporter was previously detected in LSCs from untreated AML patients [12]. We found that MRP4/ABCC4 is expressed at protein level in the CD34+CD38- KG-1a sorted population. Incomplete eradication of LSCs is likely to result in disease relapse and one approach to interfere with LSCs is to force them into differentiation. An example of the effectiveness of differentiating agents to eliminate CSCs is all-trans-retinoic acid that has been successfully used in promyelocytic AML3 subtype [29]. In addition, factors that increase cAMP levels augment the ability of this therapy to induce differentiation in blast cells [30]. Here, we evaluated differentiation in the LSC population by measuring the expression of the differentiation-specific antigens CD38 and CD11b. It is well known that the KG1a cell line fails to respond to several differentiating agents including phorbol esters, but in the present study when cAMP production was stimulated by forskolin, MRP4/ABCC4 inhibition induced CD38 expression and significantly enhanced CD11b surface expression. To our knowledge, this is the first study linking MRP4/ABCC4 to cell differentiation in a LSC population.

Furthermore, we showed *in vivo* that the administration of rolipram (PDE4 inhibitor) with probenecid (MRP inhibitor) decreased tumor growth, which induced cytostasis and apoptosis. Consistent with this pharmacological approach, similar results were obtained when MRP4 was silenced by shRNA. These findings bring up the question why the *in vivo* MRP4/ABCC4 inhibition caused cell death if there are living cells that express no MRP4/ABCC4. However, in analogy, it was shown that although most cells do not express Bcr-Abl, its blockade induces apoptosis in Bcr-Abl addicted leukemias. Thus, Bcr-Abl expression causes the accumulation of pro-apoptotic signals that are released by Bcr-Abl inhibition [31,32]. Similarly, in the present study MRP4/ABCC4 blockade increased cAMP levels resulting in cell cycle arrest and apoptosis.

The signaling pathway mediated by cAMP has emerged as a key regulator of blood cell proliferation, differentiation, and apoptosis in malignant cell populations [33]. While cAMP induces monocytic differentiation of HL-60 cells it triggers apoptosis in normal and leukemic thymocytes and in the myeloid leukemia cell line IPC-81 [6,34]. Thus, the relative balance between growth arrest, apoptosis and differentiation apparently depends on cAMP levels and the cellular context. Besides, differentiation and apoptosis can be triggered either independently or combined. We previously reported that increased cAMP levels induce differentiation in AML cell lines [13]. In the present work we show that cyclic nucleotide elevation also favored differentiation in LSCs, although it enhanced apoptosis *in vivo*. The distinct biological responses may be attributable to the different experimental models used in this study, *in vitro* versus *in vivo*. Furthermore, the fact that apoptosis could have been preceded by early differentiation in mice cannot be excluded.

The present study provides direct evidence *in vivo* and *in vitro* that MRP4/ABCC4 is involved in the regulation of LSCs differentiation as well as in tumor growth and apoptosis. In addition, clinical studies showed that MRP4/ABCC4 is highly expressed in blast cells of childhood and adult AML and higher levels of this transporter were observed in less differentiated AML subtypes [10,35]. Thus, our study, supported by these previous reports using patient samples, contributes to validate MRP4/ABCC4 as a new target for AML treatment.

Just recently, a considerable body of evidence indicates that MRP4/ABCC4 is implicated in the development of cancer, supporting its role as a novel therapeutic target [5,36]. Thus, MRP4/ABCC4 inhibition should be considered as a new alternative strategy for cancer treatment, either alone or in combination with chemotherapeutic drugs. To date, no pharmacological inhibitors have been identified that exclusively target MRP4/ABCC4. Therefore, therapeutic targeting of this transporter will require the design of potent and specific novel compounds than those available. On the other hand, a promising emerging technology to inhibit MRP4/ABCC4 is gene silencing using small interfering RNAs, including shRNA [37]. At the present time, for efficient delivery and stable knockdown of target genes in hematopoietic cells, retroviral vectors are an attractive tool [38]. In our study, a retroviral vector for MRP4/ABCC4 stable knockdown was successfully used. However, further studies are necessary to examine delivery conditions, biosafety and clinical applicability of shRNA as a biomedical tool in leukemia treatment.

## METHODS

### Animals

Six-week-old female nude (nu/nu, University of La Plata Animal Facility) mice were used. All animal care and experimental procedures complied with the Institutional guidelines and the United Kingdom Coordinating Committee on Cancer Research Guidelines for the Welfare of Animals in Experimental Neoplasia [39,40].

### Cell culture, sorting and cell differentiation

Human U937 and KG-1a leukemia cells were cultured at 37°C in a humidified atmosphere with 5% CO_2_ in RPMI 1640 or IMDM, respectively, containing 10% FCS (Invitrogen).

Fluorescent-activated cell sorting (FACS) was performed using standard protocols. Briefly, cells were incubated with mouse anti-human CD34-PE and mouse anti-human CD38-PECy5 (Beckman Coulter) for 30 min at 4°C, washed twice in PBS, analyzed and sorted using an Epics Altra Cell Sorter Flow Cytometer (Beckman Coulter). Gating on forward and side light scatter was used to exclude dead cells and debris.

In order to evaluate differentiation of the LSC population, after sorting CD34+CD38- cells were cultured in GBGM (Clear Cell Technologies) 10% FCS and treated with 10 μM forskolin (Sigma-Aldrich) and 50 μM MK-571 (Sigma-Aldrich) for 48 h.

### *In vivo* experiments

### Pharmacologic approach

The U937 cell suspension (10 x10^6^ cells) in RPMI 1640 medium was subcutaneously injected into congenitally athymic nude mice on a Swiss background (N: NIH(S)-nu) from La Plata University. When tumors were palpable the animals were randomly assigned to four groups and intraperitonelly injected every 2 days with rolipram (R, phosphodiesterase 4 inhibitor, 1.5 mg/kg), probenecid (P, MRP inhibitor, 50 mg/kg), rolipram+probenecid (R+P) or with vehicle (control, DMSO, 0.15 ml/kg) for 16 days.

### Molecular approach

This experiment was performed with U937 cells expressing short hairpin RNA (shRNA) against MRP4 (U937-shMRP4) or scrambled shRNA (U937-scramble) as previously reported [13]. Swiss nu/nu mice were injected with 10 x10^6^ cells of U937-shMRP4 or U937-scramble. A third group of mice carrying U937-scramble tumor was treated every 2 days with rolipram+probenecid (R+P, 1.5 mg/kg and 50 mg/kg, respectively) for 13 days.

In both experiments, tumors were measured using a Vernier Caliper and tumor volumes were calculated with the formula 4 × 3^−1^ × π × minor radius^2^ × major radius [41]. At the end of the experiments, animals were euthanized, and tumor samples removed and frozen at −80ºC for western blots or formalin-fixed for immunohistochemistry studies.

### Morphological studies

The morphological features of both tumor parenchyma (cellular type, grade of differentiation and growth pattern) and stroma (blood vessels, fibrosis and inflammatory infiltrate) were evaluated in hematoxylin-eosin (H&E) stained slides. Mitosis and apoptosis were counted in a 5 μ H&E stained sections by direct evaluation of 10 and 15 high power fields (HPF), respectively, using 600 x magnification, and expressed as the mean ± SEM of the percentage of the ratios between the total number of events (mitosis or apoptosis) and the total cell number per HPF. Mitotic figures were identified morphologically by the condensed “hairy” aspect of the chromosomes. Apoptosis was evaluated by identifying an eosinophilic cytoplasm, cell shrinkage with condensation, peripheral clumping or fragmentation of nuclear chromatin and by the presence of apoptotic bodies [42]. In selected tumor sections apoptosis was determined by TUNEL [terminal deoxynucleotidyltransferasa (TdT)-mediated dUTP biotin nick end labeling-technique] following manufacturer's instructions (Apoptag^®^ peroxidase in situ apoptosis detection kit, Merck Millipore, MA).

### Immunohistochemistry

Formalin-fixed, paraffin-embedded tumors were incubated with cyclin D1 (1:100, Santa Cruz Biotechnology, CA) or Bax (1:50 Santa Cruz) antibodies using the avidin-biotin-peroxidase complex technique (Vectastain Elite ABC kit, Vector Laboratories, CA). The reactions were developed with 3-3′diaminobenzidine (DAB) as described [43]. After immunohistochemistry, the specimens were lightly counterstained with 10% hematoxylin, dehydrated, and mounted.

### Isolation of membrane vesicles from U937 cell line

According to El-Sheikh et al [44], cells were harvested by centrifugation and the pellets resuspended in ice-cold homogenization buffer [0.5 mM sodium phosphate, 0.1 mM EDTA, pH 7.4] supplemented with protease inhibitors and shaken at 4°C for 60 min. Lysed cells were centrifuged at 4°C at 100.000g for 30 min, and the pellets were homogenized in ice-cold TS buffer [10 mM Tris-HEPES and 250 mM sucrose, pH 7.4] using a tight fitting Dounce homogenizer for 30 strokes. After centrifugation at 500g at 4°C for 20 min, the supernatant was centrifuged 4°C at 100.000g for 60 min. The resulting pellet was resuspended in TS buffer and passed through a 27-gauge needle 30 times. Aliquots of crude membrane vesicles were frozen in liquid nitrogen, and stored at −80°C until assayed. Protein concentration was determined by a Bio-Rad protein assay kit following the manufacturer's instructions.

### Western Blot Assay

Isolated tumors were homogenized with a polytron to obtain total fractions for Western Blot as previously described [45]. Similar amounts of protein extracts, as determined by Lowry, were loaded into each lane, separated by SDS-polyacrylamide gel and transferred to nitrocellulose membranes. The residual binding sites were blocked with 5% non-fat powdered milk in PBS containing 0.05% Tween 20, and membranes were incubated in PBS containing 0.05% Tween 20 with antibodies to p21 (1:300), Caspase-3 (1:1500) (Santa Cruz Biotechnology, Dallas, TX), MRP4 (1/50, Thermo Fisher Scientific, Waltham, MA) and α-tubulin (1:5000, Cell signaling Technology, Beverly, CA) overnight at 4 ºC. All subsequent washes were performed with the same buffer. Reactivity was developed using an anti-rabbit or anti-mouse polyclonal antibody linked to horseradish peroxidase (Vector) and developed by enhanced chemiluminescence (ECL) following the manufacturer's instructions (Amersham, GE Healthcare Lifesciences, Pittsburgh, PA).

### Flow cytometry

The characterization of the LSC population and its differentiation was evaluated by direct immunofluorescence staining using different combination of the following monoclonal antibodies: CD34-FITC, CD34-PE, CD38-PECy5, CD123-PE, CD11b-PE (Beckman Coulter). Mouse IgG antibodies were used as isotype controls.

MRP4/ABCC4 protein expression was detected by indirect immunofluorescence staining using the affinity purify polyclonal anti-human MRP4 antibody (1/50) [46]. The secondary antibody used in 1/200 dilution was goat anti-rabbit AlexaFluor 488 (Invitrogen). Incubations with antibodies were performed in PBS 2% FCS for 45 min at 4°C. Cells were washed twice with PBS 4% FCS and resuspended in PBS. Incubation without MRP4 antibody was performed as a negative control.

Data were acquired using a CyAn ADP Flow Cytometer (DakoCytomation, Beckman Coulter) and analysed using FlowJo software (Treestar).

### cAMP assay

Cells were resuspended in RPMI 1640 medium at a density of 1 × 10^6^ cells/ml and exposed to 25 μM forskolin and 1 mM 3-isobutyl-1-methylxanthine at different time points as indicated in the corresponding figure legend. Following centrifugation for 3 min at 3000 g at 4°C, 1 ml ethanol was added to supernantants (extracellular cAMP) and pellets (intracellular cAMP). Ethanol was dried and residues resuspended in 50 mM Tris–HCl, pH 7.4, 0.1% BSA for further cAMP determination. Cyclic AMP content was determined by competitive radio-binding assay for PKA using [^3^H]cAMP, as previously described [47]. The standard curve was performed using eight cAMP concentrations ranging from 0.1 to 90 pmol. Duplicate samples in at least three independent experiments were analyzed.

### Statistical analysis

Statistics were performed using GraphPad Prism 5.0 via an unpaired t test or one-way analysis of variance (ANOVA) followed by Bonferroni's comparison test. Differences were considered to be significant at p<0.05.

## SUPPLEMENTARY MATERIAL AND FIGURES



## ABBREVIATIONS

ABC: ATP binding cassette
AML: acute myeloid leukemia
CSCs: cancer stem cells
FACS: Fluorescent-activated cell sorting
FCS: fetal calf serum
FK: forskolin
GAPDH: glyceraldehyde-3-phosphate dehydrogenase
GBGM: Glycostem Basal Growth Medium
HSCs: hematopoietic stem cells
H&E: hematoxilin-eosin
HPF: high power field
IBMX: 3-isobutyl-1-methylxantine
IMDM: Iscove's modified Dulbecco's medium
LSCs: leukemic stem cells
MK: MK-571
MRPs: multidrug resistance proteins
MRP4/ABCC4: multidrug resistance protein 4/ATP binding cassette transporter 4
PBS: phosphate buffered saline
PDE4: cyclic nucleotide phosphodiesterase 4
