# Supplementary material for: Multidrug resistance protein 4/ ATP binding cassette transporter 4: a new potential therapeutic target for acute myeloid leukemia
Source: Oncotarget. 2014 Sep 2;5(19):9308–21. doi: 10.18632/oncotarget.2425 (PMC4253436; doi:10.18632/oncotarget.2425)
Supplement: Supplementary file 1 [file oncotarget-05-9308-s001.pdf]

Multidrug resistance protein 4/ ATP binding cassette transporter 4: a new potential therapeutic target for acute myeloid leukemia

Supplementary Material

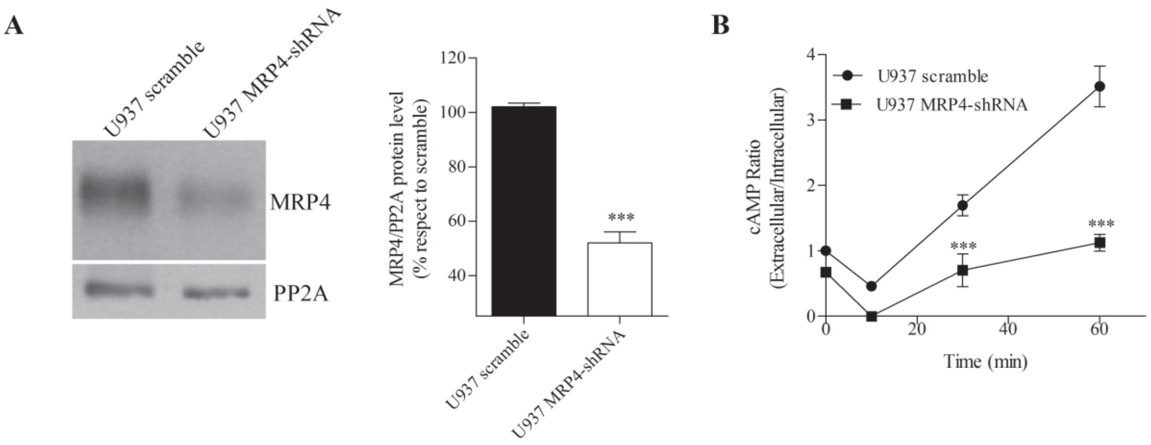

Supplementary Figure 1: Effect of MRP4 shRNA on MRP4 expression and cAMP cellular levels. (A) Representative western blot of membrane isolated from U937 cells transfected with MRP4-shRNA or scramble-shRNA. PP2A was used as a loading control. The band intensities in different western blots were quantified using ImageJ (US National Institutes of Health). MRP4 expression was significantly reduced in U937 MRP4-shRNA with respect to scramble cells. (B) Time-dependent changes in the ratio of extracellular to intracellular cAMP levels of U937 scramble and U937 MRP4-shRNA cells in the presence of 25 µM forskolin (direct adenylyl cyclase activator) and 1mM 3-isobutyl-1-methylxanthine (IBMX, PDE inhibitor). In accordance with decreased expression of MRP4, cAMP efflux also diminished in U937 MRP4-shRNA. Data represent mean±SEM (n=3). ***, p<0.001 vs. control.

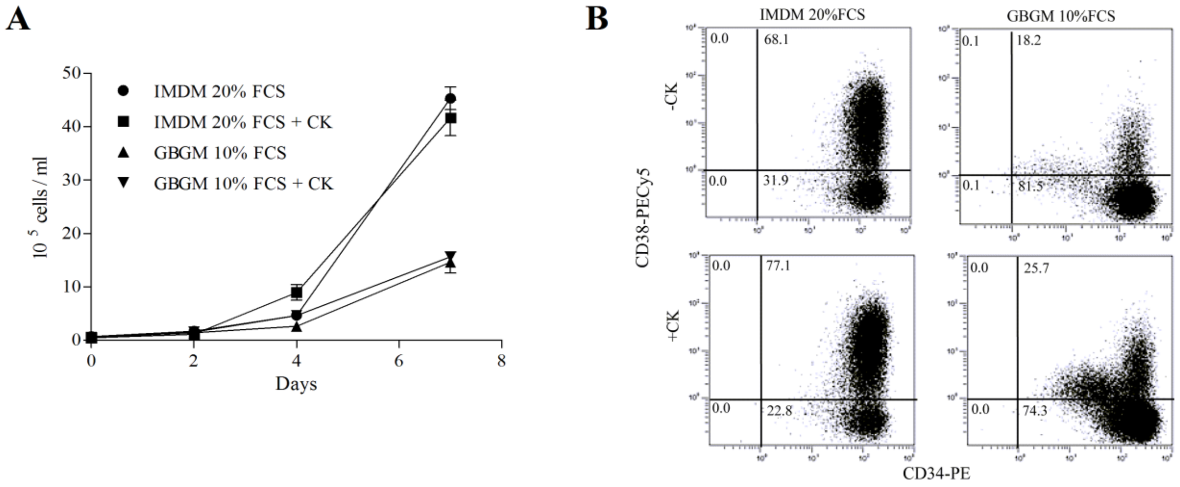

Supplementary Figure 2: LSC population retains its undifferentiated features in GBGM. (A) Proliferation curve of the LSC-sorted cells cultured in IMDM+20% FCS or GBGM+10% FCS, with or without supplements (CK: IL-3 200 ng/ml, G-CSF 200 ng/ml, SCF 200 ng/ml and FLT3 200 ng/m*l*) for 7 days. Cell proliferation was detected by cell counting and viability determination. (B) Flow cytometry analysis of CD34 and CD38 expression in the LSC population after 7 days of culturing in different media as mentioned above. Data are from one experiment representative of three.
